# Single-cell genomics technology: perspectives

**DOI:** 10.1038/s12276-020-00495-6

**Published:** 2020-09-15

**Authors:** Tae Hee Hong, Woong-Yang Park

**Affiliations:** 1 Samsung Genome Institute, Samsung Medical Center, Seoul, South Korea; 2 Department of Digital Health, Samsung Advanced Institute for Health Science & Technology, Sungkyunkwan University, Seoul, South Korea; 3 Department of Molecular Cell Biology, Sungkyunkwan University School of Medicine, Suwon, South Korea; 4 GENINUS Inc., Seoul, South Korea

**Keywords:** Medical genomics, Translational research

In the last decade, molecular and cellular technologies have evolved to give rise to the era of single-cell genomics, which allows the simultaneous measurement of thousands of genes in thousands of ‘single’ cells all at once from a single specimen. Advancements in microfluidic and molecular cloning technologies have revolutionized our understanding of complex biological processes by improving resolution to a single-cell level. Single-cell sequencing technology has also evolved over time, from processing dozens of cells to millions of cells simultaneously. New approaches to well-established models are being explored at the single-cell level in the field of medical sciences, and new rare cell types are being reported, one after another.

The Human Cell Atlas (HCA) project represents an international organized collaborative effort to develop a comprehensive reference dataset covering all cell types in the human body^1^. Functional Annotation of the Mammalian Genome (FANTOM)^2^ and Genotype-Tissue Expression (GTEx)^3^ consortia represent previous global efforts to profile the transcriptomes of various human cell types. Such public transcriptome data on multiple major organs can be used as a reference in biological studies, as they provide single-cell genomic data for mice and humans. In particular, the HCA introduced the concept of harmonization and equity in data collection and analysis, thereby promoting single-cell genomics. Ando et al. have discussed the introduction of single-cell genomic consortia that take the regional environments for developing the universal human cell reference dataset into consideration^4^.

Technical innovations in single-cell analyses continue to improve the throughput and analytical dimensions at the spatial and multi-omics level. G&T-seq^5^ and SIDR-seq^6^ represent methods for analyzing a single-cell genome and transcriptome in the same cell. CITE-seq^7^ can combine protein and transcriptome measurements into a single readout per cell. Lee et al. introduced advancements in single-cell multi-omics analysis techniques that enable us to understand genetic regulation in response to various physiological and pathological conditions^8^. In particular, Kashima et al. emphasized integrating multi-omics data from single cells and described several issues with computational methods^9^. Single-cell multi-omics analyses at the chromatin, DNA methylation and transcriptome levels during early mouse embryogenesis have provided insights into epigenetic regulation prior to cell fate decisions^10^.

Based on single-cell genome analysis techniques, we can correctly identify cell types, which is critical in the fields of developmental biology and cell therapy. The histological description of cell types will be replaced by cell typing based on the use of biomarkers and molecular profiles. Recent advances in single-cell RNA sequencing and spatial transcriptome analyses have vastly transformed our understanding of the cell types. Panina et al. focused on the current progress in the field of cell type identification^11^.

Numerous studies employ single-cell sequencing technologies in clinical samples and/or preclinical models to investigate various pathologic conditions. This series of studies provides new insights into the mechanism underlying disease and the development of therapies by deciphering intercellular heterogeneity and cellular changes that are associated with human diseases. Such a strategy for studying disease has been utilized in various clinical situations from benign conditions (e.g., inflammatory disease) to cancerous states to elucidate the link between the cellular subpopulation of interest and disease phenotypes. Quantitative molecular profiling in conjunction with investigations on the phenotypic diversity of cancer cells requires the development of computational tools. Unsupervised analyses of cell types and states underlying cancer development and progression could provide new insights into clinical outcomes. Fan et al. introduced computational methods for single-cell transcriptome data and opened new avenues for their applications in cancer research^12^. In particular, computational analysis of patients’ single-cell transcriptome data might require a robust and coherent pipeline for the applications of such methods in translational research and clinical precision medicine.

While designing clinical studies utilizing single-cell sequencing technology, researchers should consider the potential limitations associated with the acquisition and dissociation of clinical samples that frequently occur in real-world settings. For example, studies that aim to decipher the mechanisms underlying metastasis and tumor progression, which are of paramount clinical and biological importance, might require investigation of paired metastatic and primary lesion samples. However, the proper acquisition and dissociation of fresh tumor tissues for single-cell sequencing are sometimes limited due to the following: (1) as tissue acquisition occurs prior to precise pathologic diagnosis, there is uncertainty regarding whether the lesion contains the metastatic tumor of interest; and (2) minimally invasive biopsy techniques, such as percutaneous needle aspiration, are usually preferred over surgical biopsy for metastatic lesions; however, these minimally invasive techniques may not ensure enough tissue for both histologic diagnosis and single-cell sequencing. To address such issues, maintaining the tissue within the cell banker for 1‒2 weeks until the pathologic diagnosis is established can serve as a viable alternative. In addition, single nucleus sequencing in fresh frozen samples can also be considered. More importantly, a close feasibility assessment before initiating studies should be employed by interdisciplinary collaborations across physicians acquiring samples, biologists handling samples, and bioinformaticians analyzing the data.

Single-cell sequencing techniques have advanced our current understanding of human diseases. Contemporary studies using single-cell sequencing data revealed its versatility with respect to clinical aspects, such as describing tumor microenvironment landscapes, predicting treatment response (e.g., immunotherapy), discovering novel biomarkers, disease subcategorization or prognostication, monitoring residual disease after initial treatment, and deciphering the mechanisms of disease initiation and progression. However, special attention at every step, from study design to data analysis, is mandatory for fully harnessing the advantages and clinical utilities of single-cell sequencing. New technologies, such as spatial transcriptomics and CITE-seq, are expected to overcome the current limitations associated with single-cell sequencing approaches and expand the horizon in the near future. More efforts to lower the sequencing costs and mitigate the batch effects and unwanted changes introduced by tissue handling are warranted in the future to promote the application of this powerful technology in the fields of clinical diagnosis and treatment.
